# Effectiveness of First-Line Treatment with Anaplastic Lymphoma Kinase and ROS1 Protoncogene Inhibitors in Non-Small Cell Lung Cancer Patients—Real-World Evidence of Two Polish Cancer Centers

**DOI:** 10.3390/cancers17071253

**Published:** 2025-04-07

**Authors:** Michał Gil, Kinga Winiarczyk, Paweł Krawczyk, Kamila Wojas-Krawczyk, Aleksandra Łomża-Łaba, Adrian Obara, Łukasz Gajek, Katarzyna Reszka, Andrzej Tysarowski, Jarosław Buczkowski, Izabela Chmielewska, Tomasz Jankowski, Magdalena Szuba-Gil, Maciej Strzemski, Dariusz M. Kowalski, Janusz Milanowski, Maciej Krzakowski

**Affiliations:** 1 Department of Pneumonology, Oncology and Allergology, Medical University of Lublin, 20-059 Lublin, Poland; gilu.michal@gmail.com (M.G.); pawel.krawczyk@umlub.pl (P.K.);; 2 Department of Lung Cancer and Chest Tumours, The Maria Sklodowska-Curie National Research Institute of Oncology—National Research Institute, 02-781 Warsaw, Poland; kinga.winiarczyk@pib-nio.pl (K.W.);; 3 Institute of Genetics and Immunology GENIM LCC, 20-609 Lublin, Poland; 4 Department of Genetic and Molecular Cancer Diagnostics, The Maria Sklodowska-Curie National Research Institute of Oncology—National Research Institute, 02-781 Warsaw, Poland; 5 Medical Diagnostic Laboratory, University Clinical Hospital No. 4, 20-954 Lublin, Poland; 6 Department of Analytical Chemistry, Medical University of Lublin, 20-059 Lublin, Poland

**Keywords:** non-small cell-lung cancer, anaplastic lymphoma kinase, rearrangement, ALK inhibitors

## Abstract

**Introduction**: The use of ALK (anaplastic lymphoma kinase) and ROS1 (ROS1 protoncogene) inhibitors are the standard of care in advanced non-small-cell lung cancer (NSCLC) patients with *ALK* or *ROS1* gene rearrangements (approximately 5.5% of patients). Three generations of inhibitors are available, but there is no direct comparison of their efficacy in homogeneous Caucasian populations in real-world practice. In this retrospective study, we compare the efficacy of crizotinib, brigatinib, and alectinib in NSCLC patients with different clinical courses of the disease. **Materials and Methods**: One hundred four NSCLC patients with *ALK* or *ROS1* gene rearrangement were enrolled for first-line therapy with ALK inhibitors (crizotinib in 25 patients, brigatinib in 22 patients, and alectinib in 41 patients) or the ROS1 inhibitor (crizotinib in 16 patients) as part of daily clinical practice in two Polish cancer centers. Overall response rate (ORR), progression-free survival (PFS), and overall survival (OS) were compared according to treatment methods and clinical data. **Results**: In *ALK*-rearranged patients, ORR was insignificantly higher in patients treated with second-generation ALK inhibitors than in patients receiving crizotinib (68.25% vs. 48% of patients, *p* = 0.0547). Median PFS in the crizotinib group was 8 months, and in the group that received second-generation ALK inhibitors this was not reached (HR = 5.2182, 95% CI: 2.6163–10.4079, *p* < 0.0001). Similarly, median OS was significantly lower in patients treated with crizotinib than in patients receiving second-generation ALK inhibitors (26 vs. not reached, HR = 3.529, 95% CI: 1.5559–7.2258, *p* = 0.002). The efficacy of crizotinib in patients with *ROS1* and *ALK* gene rearrangement did not differ significantly (ORR—37.5 vs. 48%, median PFS—6 vs. 7 months, median OS—8 vs. 26 months, respectively). In *ALK*-rearranged patients, multivariate analysis showed that the only factor significantly increasing the risk of progression was liver metastases (HR = 2.1917, *p* = 0.0418). The risk of death was significantly higher in patients treated with crizotinib (HR = 2.4823, *p* = 0.0359) and in patients with liver metastases (HR = 3.1266, *p* = 0.0104). **Conclusions**: Second-generation ALK inhibitors are more effective than crizotinib in *ALK*-rearranged patients. Liver metastases, but not brain metastases, are the main clinical factors shortening PFS and OS in NSCLC patients treated with ALK inhibitors.

## Simple Summary

We compare the efficacy of three generations of ALK’ inhibitors in real-world practice in Caucasian population. One hundred four NSCLC patients with *ALK* or *ROS1* gene rearrangement were enrolled for first-line therapy with ALK inhibitors (crizotinib, brigatinib or alectinib). We can confirm the efficacy of second-generation inhibitors over crizotinib in the first-line treatment. No statistically significant differences were found between the effectiveness of second-generation inhibitors, but there were significantly fewer patients treated with brigatinib than with alectinib. Due to the better effect of alectinib and brigatinib on CNS metastases, liver metastases may be more difficult to treat and cause shorter PFS and OS. The favorable predictive factor was the occurrence of adverse effects of therapy. The remaining analyzed clinical and demographic factors had no effect on the efficacy of treatment.

## 1. Introduction

Lung cancer is still the leading cause of death among cancer patients in Poland and worldwide [1,2]. The most common type of lung cancer is non-small-cell lung cancer (NSCLC). Fortunately, molecularly targeted therapies can be used in molecularly predisposed NSCLC patients, which has significantly improved the prognosis in this group of patients. Moreover, immunotherapy and chemoimmunotherapy improved the prognosis in NSCLC patients who were not suitable for targeted therapy.

One of the sought-after molecular targets is abnormal ALK (anaplastic lymphoma kinase) protein, which is created as a result of *ALK* gene rearrangements. The *ALK* gene in NSCLC patients has many fusion partners, but the most common one is the *EML4* (echinoderm microtubule-associated protein-like 4) gene. As a result of such a fusion, independent activation of ALK protein is observed and constant stimulation of the cell to proliferate and grow occurs. [3,4] These genetic disorders are found in about 3–4.5% of Caucasian NSCLC patients, most often in young, non-smoking, adenocarcinoma patients in whom no other mutations have been detected in the *EGFR* or *RAS* genes. The presence of *ALK* gene rearrangement results in propensity to spread tumor cells to the CNS. In such patients, brain metastases occur in 22–33% of cases at diagnosis and could reach up to 70% of patients after crizotinib failure [5,6].

For the examination of *ALK* gene status, we can use immunohistochemical methods (IHC), fluorescence in situ hybridization (FISH), or next-generation sequencing (NGS). Each of these has advantages and limitations. The IHC method is fast and cheap to perform. It consists of staining the abnormal ALK protein using monoclonal antibodies labeled with an enzyme that breaks down the substrate into a colored product. The presence of the expression of the abnormal ALK protein is observed under an optical microscope. The FISH method uses break-apart molecular probes. These probes are labeled with red and green fluorochromes, and their fluorescence is recorded under a fluorescence microscope. Separation of the green and red signals or single red signals indicate rearrangement of the *ALK* or *ROS1* genes. On the other hand, the overlapping of these signals indicates the absence of genetic disorders. Currently, the NGS technique is most often used to diagnose abnormalities in the *ALK* and *ROS1* genes. NGS allows for the simultaneous detection of abnormalities in many genes. Gene fusions are examined in mRNA, which is transcribed into complementary DNA (cDNA). Then, gene libraries are prepared and subjected to sequencing, and the product is subjected to bioinformatics analysis [5,7].

In recent years, a number of modern drugs have been developed that inhibit tyrosine kinase of anaplastic lymphoma (tyrosine kinase inhibitors, TKIs). Three generations of ALK inhibitors (ALKis) have been developed: crizotinib (first-generation), alectinib, brigatinib, ceritinib, and ensartinib (second-generation), and lorlatinib (third-generation). Crizotinib was used in the first-line treatment or after chemotherapy failure in patients with advanced NSCLC with ALK gene rearrangements. Currently, it is used less and less often due to its poor penetration into the central nervous system (CNS) and short remission duration. Alectinib and brigatinib have replaced crizotinib in the first-line treatment of patients with advanced NSCLC. In addition, these drugs can be used in the case of resistance to crizotinib, and alectinib can also be used in the adjuvant treatment of NSCLC patients after the anatomical resection of tumors [8,9]. Ceritinib is rarely used in patients with advanced NSCLC due to frequent adverse events, which most often involve the gastrointestinal tract. On the other hand, ensartinib has not been registered by the European Medical Agency (EMA). Lorlatinib has gained the greatest potential in the treatment of patients with advanced NSCLC with *ALK* gene rearrangements [4]. This third-generation ALKi can be used in first-line therapy or in subsequent lines of treatment if there is a resistance to first- or second-generation ALKis. However, the use of lorlatinib is associated with new side effects in the form of lipid metabolism disorders, edema, neuropathy, and cognitive disorders. Importantly, the new generation of ALKis demonstrate excellent penetration into the CNS and can be used in patients with asymptomatic CNS metastases instead of brain radiotherapy [10,11,12].

*ROS1* gene rearrangement occurs in about 1–2% of Caucasian patients with NSCLC. It is caused by the fusion of the *ROS1* gene located on chromosome 6 with different fusion partners. The most common partners include the *CD74* and *EZR* genes. It leads to continuous activation of the signaling pathways (*RAF-MEK1/2-MAPK*, *STAT3-JAK*, and/or *PIK3CA-AKT-mTOR*) and causes tumor growth and metastasizing. It is usually detected using FISH, but NGS is increasingly used for this purpose [13,14,15]. The treatment of NSCLC patients with *ROS1* rearrangements is dominated by the first-generation of ROS1 inhibitor—crizotinib. However, second-generation inhibitors (entrectinib, repotrectinib) are increasingly being used, showing higher efficacy in the treatment of patients with CNS metastases [16,17,18].

The aim of this retrospective study was to determine the efficacy of ALK and ROS1 inhibitors in NSCLC patients in everyday clinical practice. The efficacy of the first-generation inhibitor (crizotinib) was compared to the effectiveness of second-generation inhibitors (alectinib or brigatinib). In addition, the efficacy of ALKis was compared between groups of patients with different clinical characteristics (e.g., depending on the presence of CNS or liver metastases or the type of genetic disorder).

## 2. Materials and Methods

### 2.1. Criteria for Including the Patients for This Study

One hundred four NSCLC patients (103 adenocarcinoma patients and one large-cell carcinoma) with *ALK* or *ROS1* gene rearrangement were enrolled for first-line therapy with ALK inhibitors or the ROS1 inhibitor as part of daily clinical practice in two Polish cancer centers: 41 patients received crizotinib (including 25 patients with *ALK* gene rearrangement and 16 patients with *ROS1* gene rearrangement), 41 patients were treated with alectinib, and 22 patients received brigatinib.

The inclusion criteria for the study (in addition to the diagnosis of rearrangements in the *ALK* or *ROS1* genes) included the following: histological or cytological diagnosis of non-squamous NSCLC; presence of lesions enabling an objective assessment of response in imaging studies using the currently applicable RECIST (response evaluation criteria in solid tumors) assessment criteria or presence of countable, non-measurable lesions; clinical stage IV (metastatic stage) or III with no possibility of radical treatment (radiochemotherapy, radiotherapy, surgery); absence of symptomatic metastases in the central nervous system or signs of progression of metastases in the central nervous system in patients after previous local treatment (surgery, radiotherapy); age over 18 years; performance status at the level of 0–2 according to the ECOG (Eastern Cooperative Oncology Group) classification; absence of clinically significant comorbidities uncontrolled by pharmacological treatment; function of the hematopoietic system, kidneys, and liver, enabling treatment with ALK or ROS inhibitors and exclusion of the co-occurrence of other malignant tumors not controlled by treatment. The method of assessing the response to treatment in all patients was high-resolution computed tomography. In almost all patients, contrast was used to visualize neoplastic lesions. Computed tomography was performed every 3 months for the first 2 years of treatment and every 6 months thereafter (for patients with persistent clinical benefits). Disease control was considered in the case of partial remission (30% reduction in the size of measurable lesions) or disease stabilization (no 30% reduction in the size of measurable lesions, but also no 20% increase in these sizes). This was in accordance with the RECIST (response evaluation criteria for solid tumors) criteria, which were also applied in the case of non-measurable lesions (individual patients).

### 2.2. Efficacy Evaluation

A CT examination was performed before starting therapy with ALK or ROS1 inhibitors. The response to treatment was evaluated using CT scans performed every 3 months, or more frequently if the progression of the disease was clinically suspected. Response to treatment was evaluated according to RECIST 1.1 [19]. Treatment was continued until disease progression or death or unacceptable toxicity. At the end of the follow-up (median follow-up of 50 months), 39 patients had not experienced progression and 60 patients were alive (censored data). Progression-free survival (PFS) and overall survival (OS) were defined as the time from the initiation of first-line treatment to progression or death.

### 2.3. ALK and ROS1 Genes Examination

The presence of abnormal ALK protein was tested by immunohistochemistry (IHC) in 13 patients. In the remaining patients, FISH (39 patients) or next-generation sequencing (NGS) in 52 patients were used to detect *ALK* or *ROS1* gene rearrangement. The D5F3 antibody clone and the Ventana test were used for IHC testing. Break-apart molecular probes labeled with fluorochromes from ZytoVision or Abbott were used for FISH testing. *ALK* or *ROS1* gene rearrangement was detected by NGS technology in the Ion Torrent S5 system with the Oncomine Focus test (Thermo Fisher; Waltham, MA, USA) or in the MiSeq system with the Archer test (Illumina; San Diego, CA, USA) [20,21,22,23].

### 2.4. Statistical Analysis

Statistical analysis was performed using Statistica 13.3 (TIBCO Software Inc., Palo Alto, CA, USA) and MedCalc (MedCalc Software Ltd., Ostend, Belgium) software 18.11.6. Kaplan–Meier survival and multiparameter Cox regression analysis were used to calculate the risk (hazard ratio, HR) of progression or death in different groups of patients. Fisher’s exact test was used to examine the associations between different clinical factors and response to therapy, the possibility of achieving one- and two-year progression-free survival, and the possibility of achieving two- and three-year overall survival. Results are presented as medians and confidence intervals (CI). A *p*-value below 0.05 was considered statistically significant.

## 3. Results

### 3.1. Characteristics of Study Population

In total, 51 women and 53 men were enrolled in the study. The mean age of the patients was 61.9 ± 11.8 years. Of the study population, 44 patients (42.3%) were smokers (mean pack-years was 25.3 ± 16.5), 57 patients (54.8%) were overweight, and the mean BMI (body mass index) in the entire group was 26.3 ± 4.7. All patients were of Caucasian ethnicity. The patients received alectinib or brigatinib at the discretion of the physician, whereas crizotinib was used earlier, before brigatinib or alectinib were reimbursed in Poland (until July 2019) and in all patients with *ROS1* rearrangement. The standard of care for patients receiving different inhibitors was the same. Detailed characteristics of the patients are presented in Table 1. Clinical and pathological data were obtained retrospectively. The research was approved by the Bioethics Committee of the Medical University of Lublin (KE-0254/160/2021).

### 3.2. Response to Treatment

In the entire study group, partial response (PR) was observed in 61 patients (58.7%), disease stabilization (SD) in 28 patients (29.9%), and disease progression (PD) in 15 patients (14.4%). In the *ALK*-rearranged group, response occurred insignificantly more frequently in patients treated with alectinib compared to patients received crizotinib (70.7% vs. 48% of the patients, *p* = 0.0648, χ^2^ = 3.14) and in patients treated with second-generation ALKis than in patients received crizotinib (68.25% vs. 48% of the patients, *p* = 0.0547, χ^2^ = 3.69). SD occurred in both groups with similar frequency (33.1% vs. 40% of the patients). Patients treated with alectinib (97.6% vs. 88% of the patients, *p* = 0.1143, χ^2^ = 2.494) and with second-generation ALKis (92.1% vs. 88% of the patients, *p* = 0.5496, χ^2^ = 0.358) showed insignificantly more frequent disease control (DC) than patients receiving crizotinib. Moreover, DC occurred insignificantly more frequently in the alectinib group than in the brigatinib group (97.6% vs. 81.8% of the patients, *p* = 0.0275, χ^2^ = 4.856). There were no significant differences in the incidence of response and disease control between patients with *ALK* and *ROS1* rearrangement treated with crizotinib (48% vs. 37.5% and 88% vs. 81.25%, respectively). The treatment response status is presented in Table 2.

### 3.3. Progression-Free Survival

In the general population, the median PFS was 19 months (95% CI: 12 to 26); 55.8% of patients had a PFS longer than 12 months and 34.6% of patients had a PFS longer than 2 years. In patients with *ALK* rearrangement, the median PFS was 24 months (95% CI: 13 to 49), whereas the median PFS in patients with *ROS1* gene rearrangements was 6 months. Information on the percentage of patients from different groups with progression-free survival at each observation point is presented in Table 2.

#### 3.3.1. Progression-Free Survival in Relation to Treatment Methods

The median PFS in patients treated with brigatinib or alectinib was not reached, while in patients receiving crizotinib it was 8 months in the *ALK*-rearranged group and 6 months in the *ROS1*-rearranged group.

**Figure 1:**
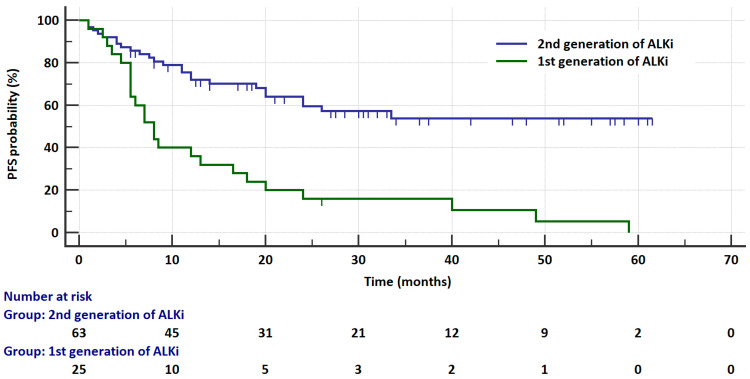
Kaplan–Meier curves that illustrate the probability of progression-free survival in ALK-rearranged patients treated with crizotinib or with second-generation ALK inhibitors.

In the *ALK*-rearranged group, the risk of progression was significantly higher in patients treated with crizotinib than in patients treated with second-generation ALKis (HR = 5.2182, 95% CI: 2.6163 to 10.4079, *p* < 0.0001, Figure 1). Moreover, the risk of progression was significantly (*p* < 0.009) higher in the crizotinib group compared to the brigatinib (HR = 3.3611, 95% CI: 1.4334 to 7.8814) or alectinib (HR = 3.4742, 95% CI: 1.716 to 7.0337) groups. The risk of progression was similar in patients receiving brigatinib and alectinib (HR = 0.9711, 95% CI: 0.4013 to 2.35, *p* = 0.9481). PFS results are presented in Table 3.

The percentage of patients with *ALK* rearrangement remaining progression-free after 1 and 2 years of follow-up was significantly lower in patients receiving crizotinib than in those receiving alectinib (χ^2^ = 7.157, *p* = 0.0075 and χ^2^ = 8.285, *p* = 0.004), and in patients receiving crizotinib compared to those receiving second-generation ALKis (χ^2^ = 5.965, *p* = 0.0146 and χ^2^ = 5.116, *p* = 0.0237). Similarly, the percentage of crizotinib-treated patients with *ROS1* rearrangement and remaining progression-free after 1 and 2 years of follow-up was significantly lower than those receiving alectinib (χ^2^ = 8.534, *p* = 0.0034 and χ^2^ = 8.884, *p* = 0.003) and second-generation ALKis (χ^2^ = 7.328, *p* = 0.007 and χ^2^ = 6.017, *p* = 0.0142). The 1- and 2-year PFS rates did not differ significantly between the crizotinib and brigatinib groups, nor between the brigatinib and alectinib groups.

#### 3.3.2. Progression-Free Survival in Relation to Clinical Factors

**Figure 2:**
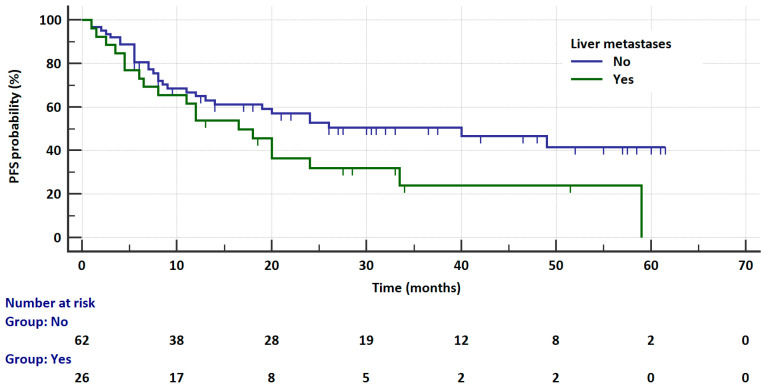
Kaplan–Meier curves showing the probability of progression-free survival in patients receiving ALK inhibitors depending on the liver metastases presence.

In the entire analyzed group, the risk of progression was not significantly related to age, gender, *ALK* or *ROS1* abnormality testing method (IHC vs. FISH vs. NGS), body weight, smoking status, size and location of tumor, CNS and bone metastases, or treatment toxicities. In the *ALK*-rearranged group, the risk of progression was insignificantly higher in patients with liver metastases compared to patients without these metastases (16.5 vs. 23 months, HR = 8204, 95%CI: 0.9623 to 3.444, *p* = 0.0655, Figure 2). The univariate analysis for PFS regarding the clinical factors in *ALK*-rearranged patients is summarized in Table 3.

**Figure 3:**
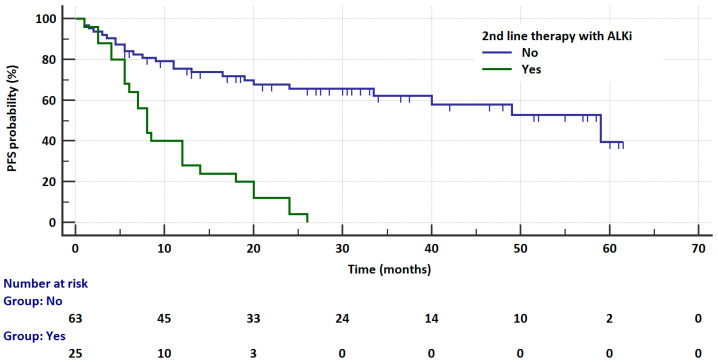
Kaplan–Meier curves showing the probability of progression-free survival in patients receiving ALK inhibitors depending on the possibility of second-line therapy application.

Patients who received second-line therapy with ALK inhibitors had a significantly shorter time to progression than patients who did not require such therapy (8 vs. 59 months, HR = 8.8073, 95% CI: 4.2259 to 18.3554, *p* < 0.0001, Figure 3). During therapy, CNS metastases developed in six (16.6%) patients treated with crizotinib (five (20%) patients had *ALK* rearrangements and one (6.25%) patient had *ROS1* rearrangements), four (18.2%) patients treated with brigatinib, and three (7.3%) patients treated with alectinib. There was no statistical significance in the frequency of secondary CNS metastases between these groups.

In the group of patients treated with second-generation ALKis, all the above-mentioned factors had no significant influence on the risk of progression. However, also in this group of patients, the median PFS was significantly lower in patients enrolled for second-line therapy with ALK inhibitors than in patients who were not given such therapy (12 vs. not reached (NR), HR = 21.1703, 95% CI: 62.83 to 71.333, *p* < 0.0001).

### 3.4. Overall Survival

In the whole examined group, the median OS calculated from the beginning of therapy was 58 months (95%CI: 28.5 to 98.0). In the entire population, 54.8% of patients survived at least 24 months and 31.7% of patients remained alive after 3 years of follow-up. In patients with *ALK* rearrangement, the median OS was 58 months (95% CI: 34 to 58 months). Information on the percentage of patients from different groups remaining alive at each observation point is presented in Table 2, and the median OS was 58 months.

#### 3.4.1. Overall Survival in Relation to Treatment Methods

**Figure 4:**
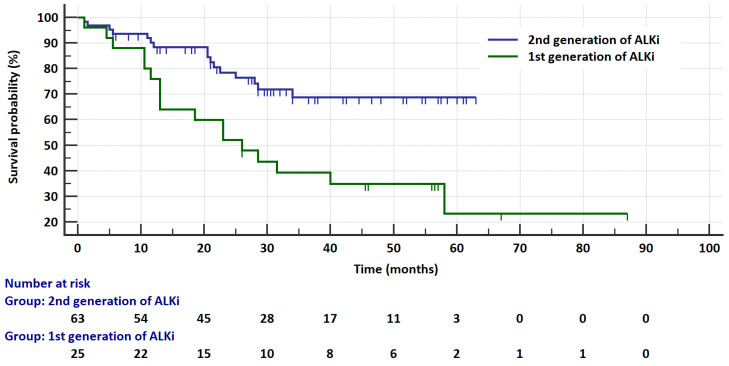
Kaplan–Meier curves that illustrate survival probability in ALK-rearranged patients treated with crizotinib or second-generation ALKis.

The median OS in patients treated with brigatinib or alectinib was not reached, while in patients treated with crizotinib it was 26 months in the *ALK*-rearranged group and 8 months in the *ROS1*-rearanged group. In patients with *ALK* rearrangements, the risk of death was significantly higher in patients treated with crizotinib than in patients treated with second-generation ALKis (26 vs. NR month, HR = 3.3529, 95% CI: 1.5559 to 7.2258, *p* = 0.002, Figure 4). In this group of patients, the risk of death was also significantly higher (*p* = 0.009) in patients treated with crizotinib than in patients treated with brigatinib (HR = 2.9219, 95% CI: 1.0568 to 8.0789) or with alectinib (HR = 2.7375, 95% CI: 1.2304 to 6.0904). The risk of death was similar in patients receiving brigatinib or alectinib (HR = 1.1323, 95% CI: 0.3722 to 3.4444, *p* = 0.8267). In patients with *ROS1* gene rearrangements, the efficacy of crizotinib in terms of the risk of death was slightly higher than in patients with *ALK* gene rearrangements (8 vs. 26 months, HR = 1.6124, 95% CI: 0.705 to 3.6876, *p* = 0.2577). Patients with ALK gene rearrangements treated with brigatinib or alectinib had a significantly (*p* = 0.003) lower risk of death than patients with ROS1 gene rearrangements who received crizotinib (HR = 0.2541, 95%CI: 0.0757 to 0.8347 and HR = 0.3082, 95% CI: 0.1084 to 0.8765, respectively).

In the *ALK*-rearranged group, there were no statistically significant differences in the percentage of patients with two- and three-year survival treated with crizotinib or second-generation ALKis. These percentages did not differ significantly between the crizotinib and brigatinib groups, nor between the alectinib groups. However, a significantly higher percentage of patients with three-year survival was observed in the alectinib group compared to the brigatinib group (χ^2^ = 8.94, *p* = 0.0028). The percentages of patients with two- and three-year survival are presented in Table 2.

#### 3.4.2. Overall Survival in Relation to Clinical Factors

**Figure 5:**
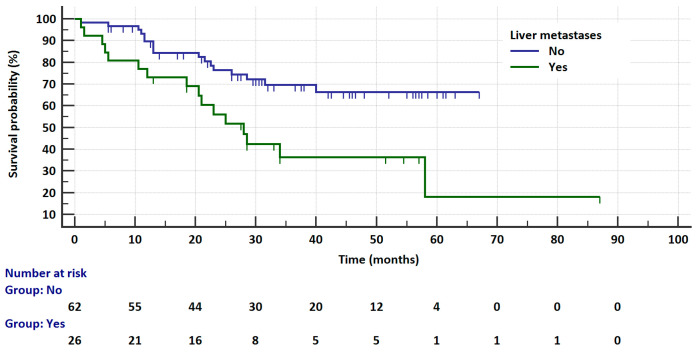
Kaplan–Meier curves that illustrate survival probability in *ALK*-rearranged patients who received ALKi depending on liver metastases.

**Figure 6:**
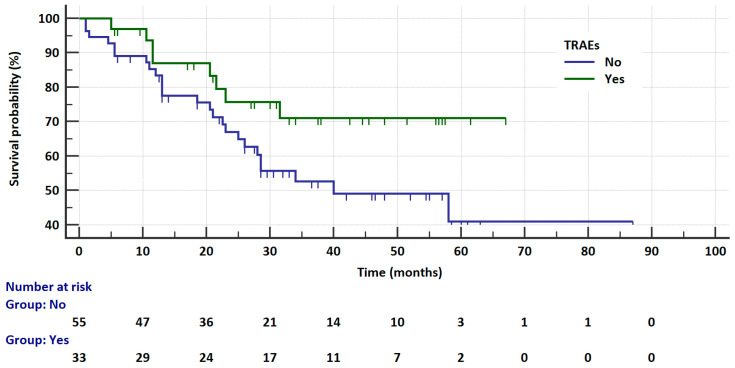
Kaplan–Meier curves that illustrate survival probability in *ALK*-rearranged patients receiving ALKi depending on treatment toxicity.

The univariate analysis for OS regarding the clinical factors in ALK-rearranged patients is summarized in Table 3. In this group, the risk of death was not significantly related to age, gender, *ALK* abnormality testing method (IHC vs. FISH vs. NGS), body weight, smoking status, location of lymph node metastases, size and location of tumor, nor to bone metastases and secondary CNS metastases. A significantly higher risk of death was observed in patients with liver metastases than in patients without such metastases (28 vs. NR months, HR = 3.2138, 95% CI: 1.4721 to 7.0165, *p* = 0.0034, Figure 5) and, insignificantly, in patients without treatment complications compared to patients with ALKi toxicity (40 vs. NR months, HR = 1.8633, 95% CI: 0.9209 to 3.7698, *p* = 0.0835, Figure 6). The median survival of patients with CNS metastases was significantly higher than in patients without such metastases (NR vs. 40 months, HR = 0.3833, 95% CI: 0.179 to 0.8205, *p* = 0.0135). However, it should be noted that 18% more patients with CNS metastases received second-generation ALKis than crizotinib (12% vs. 30.2%), which probably has an impact on the treatment results. This is related to the known advantage of second-generation ALKis over crizotinib in the treatment of CNS metastases.

The possibility of using subsequent lines of treatment with ALK and ROS1 inhibitors did not prolong the OS of examined patients. Fourteen patients with *ALK* rearrangement treated with crizotinib received second-generation ALKis in the second line of therapy (one patient received brigatinib and 13 patients received alectinib), while two patients with *ROS1* rearrangement treated with crizotinib received second-line repotrectinib in a clinical trial. Six patients treated with alectinib in the first-line and four patients receiving first-line brigatinib therapy received a subsequent line of therapy with lorlatinib. One patient progressing on alectinib received a subsequent line of therapy with brigatinib. In all 26 patients, the median PFS for the subsequent line of therapy with ALK or ROS1 inhibitors was 8 months.

**Figure 7:**
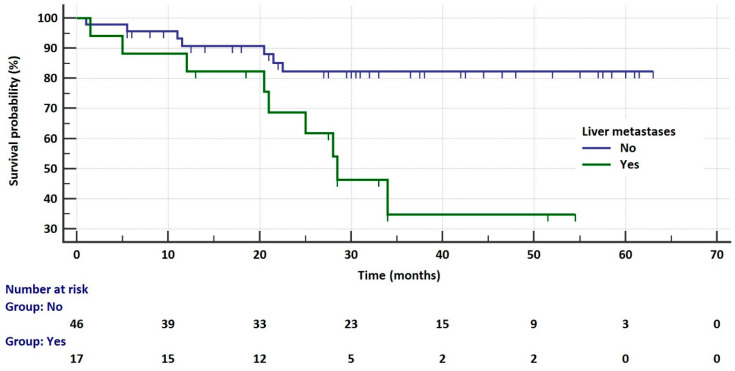
Kaplan–Meier curves that illustrate survival probability in patients who received second-generation ALK inhibitors depending on liver metastases.

In the group of patients receiving second-generation ALKis, the risk of death was independent of sex, age, body weight, smoking status, *ALK* gene abnormality diagnostic method, primary tumor size and location, lymph node metastasis, CNS and bone metastasis, and treatment complications. The risk of death was significantly higher in patients with liver metastases compared to those without such metastases (28.5 vs. NR months, HR = 5.1054, 95% CI: 1.6522 to 15.7763, *p* = 0.0046, Figure 7). Median OS did not depend on the use of ALK inhibitors in subsequent lines of treatment; however, the numbers of agents are small. The occurrence of CNS metastases during therapy with second-generation ALK inhibitors did not affect OS (however, there were only seven patients with secondary CNS metastases in this group).

### 3.5. Multivariate Risk Analysis of Progression and Death

In multivariate analysis of the *ALK*-rearranged group, crizotinib treatment (HR = 6.0311, *p* < 0.0001) and liver metastases (HR = 2.1917, *p* = 0.0418) were factors significantly increasing the risk of progression. The overall model fit was χ^2^ = 45.403 with *p* < 0.0001. The risk of death was significantly higher in patients treated with crizotinib (HR = 2.4823, *p* = 0.0359) and in patients with liver metastases (HR = 3.1266, *p* = 0.0104). However, the presence of primary brain metastases significantly reduced the risk of death (HR = 0.2258, *p* = 0.0254). In this analysis, the overall model fit was χ^2^ = 31.087 with *p* = 0.0033.

### 3.6. Treatment-Related Adverse Events

Complications related to therapy occurred in 15 (36.3%) patients treated with crizotinib, 8 (43.39%) patients treated with brigatinib, and 17 (41.5%) patients receiving alectinib. Complications were mostly grade 1 and 2 according to the CTCEA (Common Terminology Criteria for Adverse Events) criteria [24]. In the crizotinib group, hepatotoxicity occurred in six patients, nephrotoxicity in four patients, and bradycardia in two patients, and in a single patient interstitial pneumonia, visual disorders, neutropenia, and anemia was observed. In patients treated with brigatinib, increased creatine kinase (CK) concentration in serum predominated (five patients), and in a single patient, hepatotoxicity, neutropenia, rash, weakness, and weight loss occurred. In the alectinib group, hepatotoxicity occurred in nine patients, nephrotoxicity in three patients, increased CK concentration in five patients, and edema as well as anemia in a single patient. In Table 4 we summarized treatment-related adverse events in our group of patients treated with crizotinib or second-generation ALKis.

## 4. Discussion

In the observed group of 104 NSCLC patients with *ALK* or *ROS1* gene rearrangements treated with ALK or ROS1 inhibitors, we observed a median PFS of 19 months and a median OS of 58 months. In the crizotinib group, the median PFS was 8 months in patients with *ALK* rearrangements and 6 months in patients with *ROS1* rearrangements. The differences in the risk of progression and death in crizotinib-treated patients with *ALK* and *ROS1* rearrangements were not statistically significant.

The study showed that treatment with second-generation ALKis statistically significantly increases the median PFS and OS compared to treatment with crizotinib in *ALK*-rearranged patients. Patients with liver metastases showed a statistically significant reduction in PFS and OS. The presence of liver metastases is undoubtedly a poor prognostic factor in NSCLC patients. Liver metastases are resistant to both molecularly directed therapy and immunotherapy. This is due to the high vascularity of the liver and the rapid spread of cancer cells from metastatic tumors to other organs. However, the presence of primary brain metastases significantly reduced the risk of death. This is due to the fact that three times more patients with CNS metastases received second-generation ALKis rather than crizotinib. Thus, we indirectly prove the higher efficacy of second-generation ALKi compared to crizotinib in the treatment of brain metastases. Complications related to therapy occurred in 37.5% (39 patients), the most common of which were hepatotoxicity, nephrotoxicity, and increased CK activity.

The ALEX study compared the efficacy of crizotinib and alectinib in the first-line treatment of advanced NSCLC patients with *ALK* gene rearrangements. Over 300 patients were enrolled in the study, and crizotinib received 151 patients and alectinib received 152 patients. After a few years of follow-up, Mok et al. demonstrated a significant advantage of alectinib over crizotinib in NSCLC patients with *ALK* gene rearrangements. The median PFS for alectinib was 34.8 months, and for crizotinib it was 10.9 months. In our study, the median PFS for alectinib was not reached, and the median PFS for crizotinib was 8 months, and this difference was statistically significant. The median OS in this study was also significantly higher in patients treated with alectinib than with crizotinib (NR vs. 57.4 months). Similar observations were demonstrated in our analysis, with significantly longer OS for second-generation inhibitors compared to crizotinib; however, the median OS in our patients receiving crizotinib was only 26 months. The short OS in this group of patients was due to the small percentage of patients who received subsequent lines of treatment with next-generation ALK inhibitors (low rate of crossover). However, the difference in the risk of death between the crizotinib and alectinib groups was not as significant, which was related to the high crossover rate in the ALEX study. In addition, the ALEX study showed that the second-generation inhibitor is significantly more effective than crizotinib in patients with central nervous system metastases [25].

The J-ALEX study is part of the ALEX study conducted in the Japanese population of NSCLC patients with *ALK* gene rearrangements treated with alectinib or crizotinib. However, the patients in this study received half the dose of alectinib compared to the patients in the ALEX study. The obtained results are consistent with the Mok et al. observations, but the results of the J-ALEX study were not as spectacular. In the discussed study, there was also the possibility of treatment with a second-generation inhibitors in the case of progression after crizotinib. Nearly 80% of patients treated with crizotinib received alectinib after progression. This resulted in the median OS not being achieved in either study arm. However, this study also showed that alectinib treatment prolonged the progression-free survival, the time to the first CNS metastases, and inhibits disease progression in patients who have already developed such metastases [26].

In the next study, called ALESIA, the efficacy of alectinib and crizotinib was compared in the Asian population. In accordance with the ALEX study, the alectinib dose was increased to 600 mg in contrast to the J-ALEX study. The median PFS in patients receiving alectinib was 41.6 months, and in patients treated with crizotinib it was 11.1 months. A significant reduction in the risk of intracranial progression was also observed in patients treated with alectinib compared to patients receiving crizotinib [27].

The ALTA-1L study compared the efficacy and safety of crizotinib and brigatinib in first-line treatment in advanced NSCLC patients with *ALK* gene rearrangement. The investigators included Asian and non-Asian populations in the study. After follow-up, they saw a significant reduction in the risk of progression in the brigatinib group compared to the crizotinib group. In patients treated with brigatinib, the median PFS was 24.0 months in the Asian group and 24.7 months in the non-Asian group. In contrast, in these two populations, the median PFS in patients receiving crizotinib was 11.1 months and 9.4 months. Moreover, brigatinib proved to be significantly more effective than crizotinib in patients with CNS metastases [28]. The results of this study are consistent with our observations. The median PFS and the median OS in our patients treated with brigatinib was not reached, suggesting high efficacy of brigatinib.

In the next J-ALTA study, the efficacy of brigatinib in the first line of treatment or after alectinib or crizotonib therapy failure was studied in Japanese patients. In patients treated with brigatinib, the median PFS was not reached in ALKi-naive patients and was 7.3 months in patients who had previously received ALKi treatment. The authors also indicated a positive effect of brigatinib treatment on CNS metastases, as well as an extension of the time to the appearance of these metastases. Additionally, the researchers checked the effect of *ALK* gene fusion variants and the presence of *TP53* gene mutations on ALKi effectiveness. In the brigatinib group, they showed that the presence of the first variant of *EML4-ALK* fusion and lack of *TP53* gene mutations correlated with a longer PFS and improved treatment outcomes [29].

Youngkyung et al. compared the therapeutic effect of brigatinib and alectinib used in the first line of treatment in NSCLC patients with *ALK* gene rearrangement. These groups were very heterogeneous in terms of patients numbers, with 176 patients receiving alectinib and 32 patients receiving brigatinib. The authors did not demonstrate any differences in the effectiveness of both ALK inhibitors. Additionally, the researchers indicated the high safety of alectinib and brigatinib. Most side effects were mild and did not interrupt the treatment [12]. Similarly in our analysis, there were also more patients who received alectinib than brigatinib. The groups of our patients treated with alectinib and brigatinib did not achieve median PFS, and this difference was not statistically significant. The treatment was free of serious side effects.

Yang et al., in the ALTA 3 study, compared the efficacy of alectinib and brigatinib in subsequent lines of treatment after crizotinib therapy failure. The efficacy and safety of both drugs were comparable. The median PFS was 19.3 months in patients treated with brigatinib and 19.2 months in patients receiving alectinib. Although the patients in our group received second-generation inhibitors in the first line of treatment, we also obtained similar observations regarding the comparable effectiveness of both ALKis. [30].

Our group treated with crizotinib included patients with both *ALK* gene and *ROS1* gene rearrangements. Patients with *ROS1* gene rearrangements achieved a median PFS of 6 months and a median OS of 8 months. However, available clinical studies indicate better results of crizotinib therapy in patients with *ROS1* gene rearrangements. The Profile 1001 study included 53 patients with *ROS1* gene rearrangements determined by FISH. Patients received a first-generation ROS1 inhibitor—crizotinib. The median PFS was 19.3 months, while the median OS was 51.4 months [15]. Shen et al. compared the efficacy of crizotinib and chemotherapy in NSCLC patients with *ROS1* rearrangements. Patients treated with crizotinib benefited from the treatment, achieving a median PFS of 18.4 months [31]. In contrast, 56 Chinese patients with *ROS1* gene rearrangements treated with crizotinib achieved a median PFS of 23 months [32]. In another study evaluating the efficacy and safety of crizotinib, 127 patients with *ROS1* gene rearrangements received treatment, achieving a median PFS of 15.9 months [33]. Zhang et al. retrospectively searched for NSCLC patients with confirmed *ROS1* gene rearrangement and treated with first-line crizotinib. They found 168 such patients. The analysis showed a median PFS of 18 months for patients treated with crizotinib vs. 7 months for patients receiving chemotherapy. Additionally, they showed a different effectiveness of treatment in patients with different *ROS1* gene fusion partners. Furthermore, patients with CNS metastases had a statistically significantly shorter median PFS compared to patients without metastases. However, patients with these metastases had a statistically higher median PFS with crizotinib compared to chemotherapy [34].

In our study, 30 out of 104 NSCLC patients had liver metastases. In the *ALK*-rearranged group, a higher median of PFS and OS was demonstrated in patients without liver involvement (n = 62) compared to patients who had hepatic metastases (n = 26). Zhang et al. also demonstrated the influence of other clinical factors on the therapeutic effect of ALK and ROS1 inhibitors. Patients with progression due to extracranial-only metastases (including three patients with liver metastases) had a statistically lower median PFS compared to patients with intracranial-only metastases (13 vs. 23 months) [34]. This observation is consistent with our results, in which the worse prognosis is related to the deterioration of liver function by metastases.

All studies indicated the safety of first- and second-generation ALK and ROS1 inhibitors. Most side effects were mild and did not lead to discontinuation of therapy. Crizotinib most often caused increased activity of aspartate and alanine transaminases and neutropenia, as well as visual disorders [25]. Subsequently, during treatment with alectinib, increased concentration of aspartate and alanine transaminases, anaemia, nephrotoxicity, and edema were observed [12,25]. The use of brigatinib may be associated with an increased concentration of aspartate and alanine transaminases, as well as creatinine phosphokinase and anemia [12,28]. These data are consistent with our observations.

## 5. Limitations

Our study has several limitations. First, the nature of this analysis is retrospective, which could have introduced potential bias and confounding factors, but this two-center study included all consecutive Caucasian patients with NSCLC receiving ALK and ROS1 inhibitors. Furthermore, the analyzed group of patients was heterogeneous. First, the number of *ALK*-rearranged patients treated with crizotinib or brigatinib was lower than the number of patients treated with alectinib, which could be important in assessing the efficacy of this therapy. Second, patients with CNS metastases more often received second-generation inhibitors than crizotinib. Therefore, paradoxically, in the analysis of the entire cohort, these patients had a better prognosis than patients without CNS metastases. However, this proves the efficacy of treatment with second-generation ALK inhibitors in patients with CNS metastases. Third, the group of patients treated with crizotinib included patients with both *ALK* and *ROS1* rearrangements, and the efficacy of treatment in these two groups of patients did not differ significantly. A group of patients with *ROS1* gene rearrangement was included in the study due to the use of crizotinib in these patients. Crizotinib is an inhibitor of both ALK and ROS1 abnormal proteins. Clinical studies (PROFILE 1001, EUROS1, CORE) showed that crizotinib had comparable efficacy in patients with rearrangements in the *ALK* gene and in the *ROS1* gene, which was also confirmed by our observations [16,34,35]. Therefore, we decided to include the group of patients with *ROS1* rearrangements in our study. However, the cohort of patients with *ROS1* gene rearrangement is too small to draw any meaningful conclusions. Moreover, patients treated with novel ROS1 inhibitors (entrectinib, repotrectinib) were not enrolled to this study. Entrectinib was not reimbursed in Poland at the time of the study, while repotrectinib is not registered in the European Union and was available in Poland only in clinical trials (TRIDENT study). Another limitation of our study was the low number of patients undergoing second-line treatment with second- or third-generation ALKis after progression on crizotinib, alectinib, or brigatinib. This was due to the lack of immature results (patients were still treated), but more often to the deterioration of performance status and the lack of possibility of further treatment. This situation significantly affected the overall survival of the cohort of patients subjected to this retrospective study. Another problem results from the fact that the latest real-world evidence from 2023–2024 was not used in our study. Unfortunately, these data were still immature in terms of PFS. Nevertheless, the presented comparison is one of the largest studies of this type conducted in Caucasian patients treated in real-world practice with ALK and ROS1 inhibitors.

## 6. Conclusions

Summarizing our observations, we can confirm the efficacy of second-generation inhibitors over crizotinib in the first-line treatment. Due to the better effect of alectinib and brigatinib on CNS metastases, liver metastases may be more difficult to treat and cause shorter PFS and OS. No statistically significant differences were found between the effectiveness of second-generation inhibitors, but there were significantly fewer patients treated with brigatinib than with alectinib. Patients who required treatment with a subsequent generation of inhibitors had a shorter progression-free survival. Moreover, a favorable predictive factor was the occurrence of adverse effects of therapy. The remaining analyzed clinical and demographic factors had no effect on the efficacy of treatment.

## Figures and Tables

**Table 1:** Characteristics of study group.

Factors	Crizotinib Group *ALK*-Rearranged (n = 25)	Crizotinib Group *ROS1*-Rearanged (n = 16)	Brigatinib Group (n = 22)	Alectinib Group (n = 41)
**Gender**				
Female, n (%)	12 (48)	9 (56.25)	9 (40.9)	21 (51.2)
Male, n (%)	13 (52)	7 (43.75)	13 (59.1)	20 (48.8)
**Age**				
<65 years, n (%)	12 (48)	5 (31.25)	14 (63.6)	23 (56.1)
≥65 years, n (%)	13 (52)	11 (68.75)	8 (36.4)	18 (43.9)
**Smoking status**				
Smokers	16 (64)	7 (43.75)	9 (40.9)	12 (29.3)
Non-smokers	9 (36)	9 (56.25)	13 (59.1)	29 (70.7)
**Overweight**				
Yes	11 (44)	10 (62.5)	8 (36.4)	28 (68.3)
No	14 (56)	6 (37.5)	14 (63.6)	13 (31.7)
**BMI (mead ± SD)**	26.3 ± 4.1	26.7 ± 3.8	25.8 ± 5.3	27 ± 3.7
**Tumor**				
T 2–3	12 (48)	8 (50)	15 (68.2)	24 (58.5)
T 4	13 (52)	8 (50)	7 (31.8)	17 (41.5)
**Lymph nodes**				
N 0–1	4 (16)	2 (12.5)	6 (27.3)	15 (36.6)
N 2–3	21 (84)	14 (87.5)	16 (72.7)	26 (63.4)
**Primary CNS metastases**				
Yes	3 (12)	5 (31.25)	6 (27.3)	13 (31.7)
No	22 (88)	11 (68.75)	16 (72.7)	28 (68.3)
**CNS metastases during treatment**				
Yes	5 (20)	1 (6.25)	4 (18.2)	3 (7.3)
No	20 (80)	15 (93.75)	18 (81.8)	38 (92.7)
**Liver metastases**				
Yes	9 (36)	4 (25)	4 (18.2)	13 (31.7)
No	16 (64)	12 (75)	18 (81.8)	28 (68.3)
**Bones metastases**				
Yes	13 (52)	7 (43.75)	11 (50.0)	19 (46.3)
No	12 (48)	9 (56.25)	11 (50.0)	22 (53.6)
***ALK* and *ROS1* method of diagnosis**				
IHC	2 (8)	0	7 (31.8)	4 (9.7)
FISH	13 (52)	7 (43.75)	7 (31.8)	12 (29.3)
NGS	10 (40)	9 (56.25)	8 (36.4)	25 (61.0)
**Treatment-related adverse events**				
Yes	8 (32)	6 (37.5)	8 (36.4)	17 (41.5)
No	17 (68)	10 (62.5)	14 (63.6)	24 (58.5)
**Second-line therapy with ALK or ROS1 inhibitors**				
Yes	14 (56)	2 (12.5)	4 (18.2)	7 (17.1)
No	11 (44)	14 (87.5)	18 (81.8)	34 (82.9)

T—tumor, N—node, CNS—central nervous system, IHC—immunohistochemistry, FISH—fluorescence in situ hybridization, NGS—next-generation sequencing, SD—standard deviation.

**Table 2:** Response to treatment, progression-free survival, and overall survival rates by treatment method.

Factors	Crizotinib Group *ALK*-Rearranged (n = 25)	Crizotinib Group *ROS1*-Rearanged (n = 16)	Brigatinb Group (n = 22)	Alectinib Group (n = 41)
**Response to treatment**				
PR, n (%)	12 (48)	6 (37.5)	15 (68.2)	29 (70.7)
SD, n (%)	10 (40)	7 (43.75)	3 (13.6%)	11 (26.9)
PD, n (%)	3 (12)	3 (18.75)	4 (18.2%)	1 (2.4)
Disease control, n (%)	22 (88)	13 (81.25)	18 (81.8)	40 (97.6)
**1-year PFS ratio, n (%)**	10 (40)	5 (31.25)	13 (58.1)	30 (73.2)
**2-year PFS ratio, n (%)**	5 (20)	2 (12.5)	6 (27.3)	23 (56.1)
**2-year OS ratio, n (%)**	13 (52)	6 (37.5)	9 (40.8)	29 (70.7)
**3-year OS ratio, n (%)**	9 (36)	3 (18.75)	2 (9.1)	19 (46.3)

PR—partial response, SD—stable disease, PD—progressive disease, PFS—progression-free survival, OS—overall survival.

**Table 3:** Univariate analysis for PFS and OS in *ALK*-rearranged patients treated with different ALK inhibitors.

Characteristics	N (%)	Median PFS (Months)	HR (95% CI) *p*	Median OS (Months)	HR (95% CI) *p*
**Age**			0.7915		1.3731
<65 years	49	26	(0.4451–1.407)	58	(0.6831–2.76)
≥65 years	39	19	0.4259	NR	0.3734
**Gender**			0.782		1.0685
Male	47	26	(0.4416–1.3847)	58	(0.5368–2.1268)
Female	41	14	0.3989	NR	0.8504
**Overweight**			1.1039		0.9896
No	41	20	(0.6199–1.9656)	NR	(0.4964–1.9727)
Yes	47	24	0.7371	58	0.9763
**Smoking**			0.6494		1.0416
No	51	33.5	(0.363–1.1616)	58	(0.5189–2.0907)
Yes	37	19	0.1456	NR	0.9087
**Tumor**			1.1758		1.4202
T2–3	44	20	(0.6633–2.084)	58	(0.7071–2.8525)
T4	44	24	0.5793	NR	0.3241
**Lymph nodes**			0.6285		0.9791
** N1**	25	NR	(0.3379–1.1690)	NR	(0.4535–2.1142)
** N2–3**	63	20	0.1425	58	0.9572
**CNS metastases**			0.6007		**0.3833**
Yes	66	59	(0.3205–1.1258)	NR	**(0.179–0.8205)**
No	22	19	0.11118	40	**0.0135**
**Liver metastases**			1.8204		**3.2138**
Yes	26	16.5	(0.9623–3.444)	28	**(1.4721–7.0165)**
No	62	40	0.0655	NR	**0.0034**
**Bones metastases**			0.8515		1.4523
Yes	43	33.5	(0.4832–1.5008)	NR	(0.7267–2.9024)
No	45	20	0.5783	NR	0.2908
**Treatment**			**5.2182**		**3.3529**
Crizotinib	25	8	**(2.6163–10.4079)**	26	**(1.5559–7.2258)**
Second-generation ALKi	63	NR	**<0.0001**	NR	**0.002**
**Treatment**			0.9711		0.8831
Brigatinib	22 (34.9)	NR	(0.4013–2.35)	NR	(0.2903–2.6864)
Alectinib	41 (65.1)	NR	0.948	NR	0.827
**Second-line therapy with ALKi**			**8.8073**		1.6782
yes	25	8	**(4.2259–18.3554)**	31.5	(0.8002–3.5195)
no	63	59	**<0.0001**	NR	0.1707
**TRAE**			0.754		0.5367
Yes	33	24	(0.4219–1.3476)	NR	(0.2653–1.0858)
No	55	20	0.3406	40	0.0835

PFS—progression-free survival, OS—overall survival, HR—risk ratio, CI—confidence interval, ALKi—ALK inhibitor, CNS—central nervous system, NR—not reached, TRAE—treatment-related adverse event.

**Table 4:** Treatment-related adverse events (TRAE) in NSCLC patients treated with crizotinib or second-generation ALKis.

Factors	Crizotinib Group (n = 41)	Brigatinib Group (n = 22)	Alectinib Group (n = 41)
Any TRAE, n (%)	15 (36.3%)	8 (43.4)	17 (41.5)
Grade 3 o4 TRAE, n (%)	1 (2.4)	1 (4.5)	2 (4.9)
Hepatotoxicity, n (%)	6 (14.6%)	1 (4.5)	9 (21.95)
Nephrotoxicity, n (%)	4 (9.8)	-	3 (7.3)
Pneumonia, n (%)	1 (2.4)	-	-
Bradycardia, n (%)	2 (4.9)	-	-
Visual disorders, n (%)	1 (2.4)	-	-
Neutropenia, n(%)	1 (2.4)	1 (4.5)	-
Anemia, n (%)	1 (2.4)	-	1 (2.4)
Increased CK concentration	-	5 (22.7)	5 (12.2)
Rash, n (%)	-	1 (4.5)	
Weakness and weight loss, n (%)	-	1 (4.5)	-
Edema, n (%)	-	-	1 (2.4)

## Data Availability

The data presented in this study are available in this article.
